# Cancer Center Recommendations to Mitigate COVID-19 Impact in Patients With Cancer: Low-Resource Settings Version

**DOI:** 10.1200/GO.20.00093

**Published:** 2020-04-13

**Authors:** Luis Pino, Carlos Perez, Andres Cardona, Ivan Triana

**Affiliations:** Luis Pino, MD, MSc, MBA, Carlos Ardila Lülle Cancer Institute, Fundación Santafe, Bogotá, Colombia; Carlos Perez, MD, Clínica de Marly, University Hospital La Samaritana, Bogotá, Colombia; Andres Cardona, MD, PhD, MSc, Clínica del Country, Bogotá, Colombia; and Ivan Triana, MD, Carlos Ardila Lülle Cancer Institute, Fundación Santafe, Bogotá, Colombia

## TO THE EDITOR:

Liang et al^1^ described a Chinese cohort of patients with cancer during the COVID-19 outbreak. The clinical characteristics and outcomes were relevant to our clinical practice. As a whole, patients with cancer have a higher risk for severe events (39% *v* 8%; *P* = .0003), including death. Outcomes are even worse in patients who received active treatment in the first month before infection (75% *v* 43%; odds ratio, 5.34). These patients also had a faster evolution to deterioration (13 *v* 43 days; *P* = .0001).

These data are relevant for cancer institutions. In the COVID-19 scenario, and especially in low- and middle-income settings, prioritization of adequate pathways for these patients is critical.

Liang et al^1^ described only 18 cases (1% of the entire COVID-19 population). We have limited data to make deeper conclusions, but in an attempt to help cancer centers in low-resource settings we have compiled^2-4^ and created some adjusted recommendations:

1. Social containment is the key. Cancer centers must move to virtual assistance through technological platforms to give telemonitoring and tele-assistance, especially to controlled and older patients, to ensure they stay at home.^4^ This clinical procedure must be in accordance with a virtual administrative process. There are some free platforms that can be used, such as Google Suite or Skype.
2. Tumor boards and scientific meetings must move to virtual modalities, decreasing the volume of cases to present to optimize the attending time for COVID-19–positive patients.
3. Prioritize and switch noncurative medical and surgical treatments. This means a change of immunotherapy schedules to 4 weeks (nivolumab, pembrolizumab) or even 6 weeks (pembrolizumab off-label) for selected patients. Switch to oral therapies for advanced cases with intravenous treatments. Temporarily discontinue noncritical therapies, such as bisphosphonates or denosumab, and optimize devices such as on-body injector pegfilgrastim to avoid the return of the patients the next day to the clinic.
4. Use strict selection of patients for in-hospital chemotherapy. This must be offered only to curative-intent treatments with higher-toxicity combinations (acute leukemias, high-grade lymphomas, soft tissue sarcomas).
5. Give long-term (6 months) formulations to patients in long-term oral treatment, such as hormonal therapies. For patients with prostate cancer using oral therapies, the follow-up can move to every 2 months.
6. All patients with cancer attending the cancer center must enter into a COVID-19 protocol to measure body temperature before entrance and to initiate the diagnostic process in case of symptoms. Cancer centers must have COVID-19 diagnostic tests on site.
7. Optimize protective measures for the health care team, improving use of surgical gowns instead of civilian clothes and using a strict protocol for disinfection of personal devices.
8. It is critical to standardize with the infectious disease, bioethics, and intensive care unit departments the entry criteria of patients with cancer according to their prognosis. Patients and their families must be informed about institutional protocols to step down medical interventions and prioritize support and palliative care in-house.
9. In hospices, where available, this infrastructure can be adapted as expansion for critical oncological care for selected patients who will be treated according to protocols and entry criteria.
10. Create a population registry of this cohort of COVID-19–positive patients with cancer to define clinical characteristics, disease dynamics, response to therapies, and outcomes that can enhance data about this special group of patients and refine institutional protocols.
11. Where available, cancer centers must create a digital platform to enhance patient, caregiver, health authority, and health care team integration; data and information flow; and even telemedicine to accomplish the previous activities in a better way.

This is a nonpublished scenario with scarce data. Cancer institutions and our patients must co-create responses to deal with the pandemic in a better way. Low-resource settings in cancer must have prioritization and compassionate care as the axis of their strategy.
